# Oxidant and antioxidant status in children with juvenile idiopathic athritis

**DOI:** 10.1186/1546-0096-9-S1-P296

**Published:** 2011-09-14

**Authors:** Hakan M Poyrazoglu, Ikbal Gökçek, Cevat Yazici, Ruhan Düsünsel, Zübeyde Gündüz, Ismail Dursun

**Affiliations:** 1 Erciyes University Medical Faculty, KAYSERI, Turkey

## Background

Although the pathogenesis of juvenile idiopathic arthritis is poorly understood, it is thought that a triggered inflammation by neutrophil activation due to any reason, released free oxygen radicals and insufficiency of antioxidant system may play role in the chronic course of this disease.

## Aim

In present study, it was aimed to identify the status of oxidant-antioxidant systems.

## Methods

In this study, 45 patients with JIA were included. In patients with active disease and in remission, erythrocyte sedimentation rate (ESR), C-reactive protein (CRP), Advanced Oxidation Protein Products (AOPP), myeloperoxidase (MPO), thiol, Total Anti-oxidant capacity (TAC), Total Oxidant Capacity (TOC) and neopterin levels were measured.

## Results

Mean age of patients was 9.6 years. Female: male ratio was 1.5 (F/M=27/18). Oligoarticular JIA was diagnosed in 21 patients, whereas polyarticular JIA and systemic JIA in 17 and in seven patients, respectively. During study period, 23 patients had active disease, while 22 patients were in remission. Mean duration of disease was 2.2 years.

Serum ESR, CRP, TOC, TAC and, MPO levels were found higher in the patients with active disease than those in remission (p<0.05). No difference was found in neopterin, AOPP and thiol levels.

## Conclusion

The results of the present study indicate that oxidant-antioxidant systems are active in JIA patients during active phase. This activation in favor of oxidants is interesting. More comprehensive studies are needed to determine whether this is cause or result.

